# Traumatic Cataract in Children in Eastern China: Shanghai Pediatric Cataract Study

**DOI:** 10.1038/s41598-018-20982-1

**Published:** 2018-02-07

**Authors:** Yu Du, Wenwen He, Xinghuai Sun, Yi Lu, Xiangjia Zhu

**Affiliations:** 1 Department of Ophthalmology, Eye and Ear, Nose, and Throat Hospital of Fudan University, 83 Fenyang Road, Shanghai, 200031 China; 2 Key Laboratory of Myopia, Ministry of Health, 83 Fenyang Road, Shanghai, 200031 China; 3 Eye Institute of Eye and Ear, Nose, and Throat Hospital of Fudan University, 83 Fenyang Road, Shanghai, 200031 China; 4 Key Laboratory of Visual Impairment and Restoration of Shanghai, Fudan University, 83 Fenyang Road, Shanghai, 200031 China

## Abstract

Traumatic cataract is a main cause of visual impairment in pediatric populations and is preventable. Awareness of the causes and consequences of pediatric eye trauma play roles in health education and prevention of blindness. We conducted a retrospective chart review based on 5-year clinical data of pediatric traumatic cataract cases treated at the Eye and Ear, Nose and Throat Hospital of Fudan University, Shanghai, China. Clinical features including demographic data, injury mechanism, and causative agents were analyzed. A total of 321 eyes of 321 children (male, 74.1%) were included. Penetrating injury accounted for 76.6% of all injuries; 65% of children with such injuries underwent their first surgery on the day of injury. The average age at injury was 6.3 ± 3.7 years, while the peak age was from two to eight years. The main causes of ocular injury were sharp metal objects, toys, and wooden sticks. The causative agent changed with increasing age; however, scissors were the leading cause within almost all age groups. Cataract surgery significantly improved visual acuity in children with traumatic cataract. These findings add information regarding the characterization of pediatric traumatic cataract in China and can help guide safety education and preventative measures.

## Introduction

Traumatic cataract accounts for a large proportion of monocular visual disability and blindness in pediatric populations, especially in developing countries^1–4^. Despite great advancements in diagnostic and treatment methods, traumatic cataract complicated with injuries such as corneal perforation, lens luxation, secondary glaucoma, endophthalmitis, and retinal detachment can still result in permanent visual impairment, even vocational disabilities, in many patients^5,6^. To prevent such serious conditions, evidence-based information on the causes of these injuries and education as well as prevention measures are of crucial importance.

Studies on traumatic cataract in children have been reported from developing countries such as India and Nepal; however, studies on traumatic cataract in children in China, the most populous country, are limited. The only previous report on the demographic features of traumatic cataract in Chinese children was conducted in 2012 involving 117 patients in Shandong Province^7^, which is far from sufficient to understand this condition.

In this study, we retrospectively analyzed mechanical ocular injuries in children presenting with cataracts who were admitted to the Department of Ophthalmology, Eye and Ear, Nose and Throat Hospital of Fudan University, Shanghai, China, based on 5-year single-center experience of 321 cases, aiming to address gaps in the current knowledge of injury-induced traumatic cataract in children in China. The Department of Ophthalmology, Eye and Ear, Nose and Throat Hospital of Fudan University is one of the top eye centers in China, and the patients admitted to our hospital come from various provinces of eastern China, including Zhejiang, Jiangsu, Anhui, and Fujian. The hospital has ~800,000 outpatient visits and admits an estimated 15,000 patients per year; thus, the demographics of pediatric patients with ocular injury at our hospital are likely representative of the general situation in eastern China.

## Results

### Baseline Information

A total of 321 eyes of 321 patients were included during the 5-year study period; all of these eyes had been injured unilaterally. The patients included 238 (74.1%) boys and 83 (25.9%) girls, with a sex ratio of 2.9:1. The average age at injury was 5.8 ± 3.9 years for the girls and 6.5 ± 3.6 years for the boys. Penetrating injuries accounted for 76.6% (246/321) of all injuries, with a sex ratio as high as 2.9:1, similar to that for blunt injuries (2.8:1).

### Distribution of Children with Traumatic Cataract by Age

**Figure 1:**
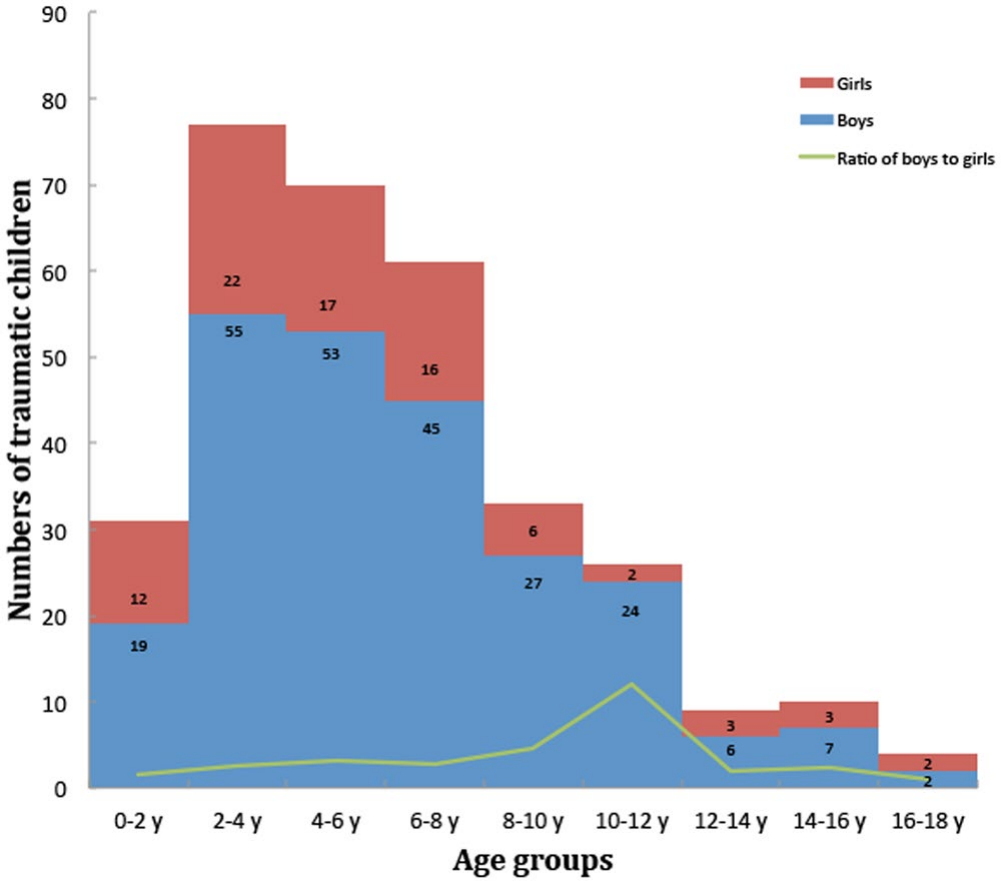
Distribution of traumatic cataract in boys and girls among different age groups.

As shown in Fig. 1, the age distribution of the traumatic cataract cases at the time of injury was skewed to the right. The majority (24.0%) of patients were 2–4 years old, 21.8% were 4–6 years old, and 19.0% were 6–8 years old. The proportion of boys was higher than that of girls in almost every age group and was highest in the 10–12 year age group. After 12 years of age, the ratio of boys to girls decreased from 12:1 to 1:1.

### Interval between Injury and the First Surgical Intervention

Children with traumatic cataract caused by penetrating injuries underwent surgical treatment within a median of 0 days (range: 0–13 years) after admittance to our hospital. Sixty-five percent (160/246) arrived at our hospital on the same day of the injury. However, for those with blunt injuries, this interval was much longer, ranging from 0 days to 11 years, with a median of 3 months.

### Causes of the Ocular Injuries

Table 1 lists the causes of injuries identified in our patients. The main causes were sharp metal objects (40.2%), toys (16.5%), and wooden sticks (11.2%). Of the sharp metal objects, scissors, knives, and needles caused 67.4% (87/129), 17.8% (23/129), and 10.08% (13/129) of the eye injuries, respectively. The most frequent toys inducing ocular injury were toy bullets (45.3%, 24/53), plastic toys (37.7%, 20/53), and slingshots (15.1%, 8/53). Pencils were the leading causative agent among the stationery items (88.0%, 14/16). Nine children were unable to recall the cause of their injury, and all suffered from blunt injuries. Sharp metal objects were the leading causative agent for traumatic cataract in children within the various age groups, for both girls and boys. The top two causative agents of traumatic cataract for boys and girls in each age group are listed in Table 2.

**Table 1:** Causes of injuries leading to traumatic cataract in children.

Cause of injury	Number	Total	Percentage (%)
Sharp metal object: scissors/needle/knife/sheet metal	87/13/23/6	129	40.2
Toy: bullet/plastic toy/slingshot/kite string	24/20/8/1	53	16.5
Stick: wooden/bamboo	36	36	11.2
Sharp non-metal object: glass/ceramic	18/4	22	6.9
Firecrackers	19	19	5.9
Stationery item: pen(cil)/book/paper	14/1/1	16	5.0
Stone	10	10	3.1
Hit	10	10	3.1
Fall	4	4	1.3
Car accident	4	4	1.3
Lighter explosion	2	2	0.6
Spring	2	2	0.6
Nail	1	1	0.3
Plug	1	1	0.3
Wasp	1	1	0.3
Shrimp shell	1	1	0.3
Animal bone	1	1	0.3
Not clear	9	9	2.8

**Table 2:** The top two causative agents of traumatic cataract in each age group.

Age group (years)	Causative agent			
	Girls		Boys	
	First	Second	First	Second
0–2	Scissors	Ceramics	Scissors	Iron nail
2–4	Scissors	Toy bullet	Scissors	Needle
4–6	Scissors	Firecracker	Scissors	Toy bullet
6–8	Scissors	Stone	Scissors	Pencil
8–10	Scissors	Stone	Toy bullet	Firecracker
10–12	Scissors	/	Plastic toy	Scissors/Glasses
12–18	Scissors	Pencil	Wood stick	Fist

### Structural Injuries Associated with Traumatic Cataract

Table 3 displays the structural injuries occurring concomitantly with traumatic cataract. Of the 246 cataractous eyes with penetrating injuries, 97.6% (240/246) were inflicted with corneal perforation and 23.2% (57/246) with iris injuries including anterior and posterior iris synechia and iridodialysis. Of the cataractous eyes with blunt injuries, the iris was the most frequently affected structure (50.7%, 38/75), and lens luxation was the second most frequent concomitant injury, accounting for 20% (15/75) of the penetrating injuries.

**Table 3:** Structural injuries associated with traumatic cataract.

Associated injury	Penetrating injury		Blunt injury	
Corneal perforation	240	(97.6%)	0	(0.0%)
Iris synechia/iridodialysis	57	(23.2%)	38	(50.7%)
Lens luxation	4	(1.6%)	15	(20.0%)
Vitreous hemorrhage	19	(7.7%)	2	(2.7%)
Choroidal detachment	4	(1.6%)	0	(0.0%)
Retinal detachment	11	(4.5%)	2	(2.7%)

### Visual Acuity Before and After Cataract Surgery

As listed in Table 4, 66.3% (163/246) of children with cataracts caused by penetrating injuries cooperated in both the preoperative and postoperative examinations of visual acuity, and 86.7% (65/75) of those with cataracts caused by blunt injuries cooperated in both examinations. Among these children, 90.8% (148/163) of those with cataracts caused by penetrating injuries presented with an uncorrected visual acuity (UCVA) worse than 0.1, while this proportion was significantly reduced to 34.4% (56/163, P < 0.001, chi-square test) after cataract surgery. Among the children with cataracts caused by with blunt injuries, 72.3% (47/65) had an UCVA worse than 0.1, while this proportion was significantly reduced to 13.9% (9/65, P < 0.001, chi-square test) after cataract surgery. Both preoperatively and postoperatively, higher proportions of children with traumatic cataract caused by blunt injuries were found to have a visual acuity better than 0.1, compared with those with penetrating injuries (P = 0.001, chi-square test). As displayed in Table 4, postoperatively, the majority of children had a visual acuity range of 0.1–0.3 in the penetrating injury group (41.1%) and ≥0.3 in the blunt injury group (50.8%).

**Table 4:** Visual acuities of children with traumatic cataract before and after cataract surgery according to the type of injury.

Visual acuity	Penetrating injury				Blunt injury			
	Preoperative		Postoperative		Preoperative		Postoperative	
<0.1	148	(90.8%)	56	(34.4%)	47	(72.3%)	9	(13.5%)
≥0.1 to <0.3	13	(8.0%)	67	(41.1%)	17	(26.2%)	23	(35.4%)
≥0.3	2	(1.2%)	40	(24.5%)	1	(1.5%)	33	(50.8%)
Total	163	100%	163	100%	65	100%	65	100%

## Discussion

Pediatric ocular injuries may cause significant visual impairment and psychological stress in children, as well as their guardians, and significant economic burden in developing countries such as China. Traumatic cataract is one of the most frequent morbidities caused by ocular injury^8^. Even with qualified first aid and surgical treatment, many children with traumatic cataract end up with permanent visual disabilities such as amblyopia or even blindness because of the severity of the injury^9^. In traumatic cataract cases, prevention is always better than treatment. Adult supervision, especially in situations involving potential injury-causing objects, can effectively prevent ocular injuries^10^. In the present study, we aimed to summarize our clinical experience with treating mechanical traumatic cataract at the largest eye center in eastern China over a 5-year period, and to evaluate the demographics and etiologies associated with these cases to provide evidence-based knowledge for health awareness education.

Our study included children less than 18 years of age with traumatic cataract. In almost all of the age groups, traumatic cataract occurred predominantly in boys, which was consistent with previous studies from other developing countries, such as Malaysia^3^, Nepal^11^, and India^2^. In Australia, Staffieri *et al*. also found a higher prevalence of pediatric traumatic cataract in boys^12^. The overall sex ratio in our study was 2.9:1 (238 boys and 83 girls), higher than the sex ratio of 1.18:1 from the latest available census data in China. This high sex ratio could be explained by the difference in the nature of boys versus girls and by the greater inclination of boys to play with potentially dangerous objects, such as scissors, sticks, and firecrackers.

Among the different age groups of children with traumatic cataract, the majority of ocular injuries occurred at 2–8 years of age, accounting for almost two-thirds of all children recruited in our study. This is in line with the findings of Batur *et al*., who found that open globe injury in Turkish children occurred most frequently in the 3–7 year age group^13^. This is possibly because children of this age are able to walk independently, and their guardians are not always close by, resulting in insufficient supervision. In addition, children of this age are very curious and eager to explore their external environment, yet with limited awareness of danger. Despite the inadequate ability to self-protect, children eventually learn the concept of danger through daily observation; thus, with the age, the occurrence of ocular injury appears to decline, as shown by our data. The marked decrease in frequency of traumatic cataract observed in children older than 8 years may be attributed to schooling. Regular presence at school not only reduces their time participating in outdoor activities and hence their exposure to dangerous items, but also allows children to receive systemic safety education. In addition, due to the different nature of boys and girls, boys of all age groups, even adults, tend to experience more injuries than do girls, which is consistent with most of the studies conducted in other countries^4,14^. In our study, the ratio of boys to girls peaked at 12:1 around 10–12 years old and then decreased to 1:1 at 18 years. A possible explanation is that boys mature later than girls in most cases. With a tendency to be more active and naughty, boys spend less time sitting at a desk to learn and thus are more susceptible to injuries such as those related to sports. However, as a result of safety awareness education, boys suffer from ocular injuries less frequently as they grow older.

Causes of ocular injuries are diverse and tend to vary among different countries as a result of different socioeconomic backgrounds and living environments^15^. In predominantly agrarian countries such as northern India^2^, the most common injury-causing agents leading to traumatic cataract are thorns and stones. In suburban Malaysia^3^, organic foreign bodies were the most common agents causing eye injuries among children with traumatic cataract, as reported by Adlina *et al*. Sharp pointed objects including scissors and pens were found to be the most common causes of injury in Australian children^12^. In our study, the majority of children with traumatic cataract in eastern China suffered from ocular injuries caused by sharp metal objects, of which scissors accounted for 67.4% of the total injuries. Toys including toy bullets and plastic toys were the second leading causative agent; Xu *et al*. reported that botanic materials were the second most common causes of eye injuries in a population in Shandong Province of China^7^. Of note, scissors are the leading injury-causing agent in the majority of Chinese children, both boys and girls, in all age groups in northern and southern regions. Therefore, preventing children from playing with scissors is likely to prevent pediatric cases of traumatic cataract significantly. Caregivers should always be careful in providing scissors to children; dull-tip scissors should be the first choice for use with children.

The second most common causative agent seems to vary according to the physical development of children. For children less than 2 years of age, sharp objects, such as ceramics in girls and iron nails in boys, were the second most common causative agents. At older ages, children play independently in outdoor activities and with toys, and firecrackers and toy bullets became common causative agents. Children of elementary school age become more aware of these obviously dangerous objects; at these ages, stationery tools were more common injury-causing objects for Chinese children, who spend most of their time at school, where there is less environmental risk of ocular injury. Of special note, although ocular injuries became less frequent with age, injuries caused by fistfights gradually increased in frequency, especially in boys.

Although an important public health concern, most childhood injuries are preventable^16^. In addition to educating children about potentially injury-causing objects, most current prevention efforts in China have been focused on the childcare provided by parents or other caretakers. It is almost inevitable that children get out of their caretakers’ sight, and Cusinamo *et al*. reported that the vast majority of injuries occurring at home are unwitnessed by adult caregivers^17^. Thus, a safe environment with the least potential for risk is essential to prevent childhood injuries. Penetrating injuries are always caused by obviously dangerous objects such as scissors, nails, and needles. Usually caretakers are sensitive to sharp-pointed objects and fully aware of the importance of keeping these objects out of children’s reach. However, as found in our study, most blunt injuries in children were caused by normal items such as plastic toys, wooden sticks, and ceramics. Winkler *et al*. developed a list of potentially dangerous children’s toys in America based on narratives from injured children or their parents and found that this list differed distinctly from a conventional dictionary of dangerous toys^18^. Accordingly, to prevent toy-related injuries effectively, such lists for different regions should be developed for targeted surveillance of children’s toys regarding safety. Furthermore, parents should pay attention to the placement of home decorations, such as vases and chinaware. Higher stands and those bolted to walls may be safer choices to place these decorations.

When an accident occurs, effective first aid and timely surgical intervention are of great importance^19^. Due to the distinct features of ocular injuries, children with penetrating injuries tend to experience a more obvious deterioration in visual acuity^16^, making it easier for parents to notice the penetrating injury and take the child to the hospital at that time. However, the onset of traumatic cataract due to blunt injury tends to be gradual, which might delay proper treatment and result in an unsatisfactory prognosis in many children^20^. In children who mention being hit in the eye, regardless of whether there is immediate vision loss or wounds around the eyeball, parents should closely observe the child’s eye for timely detection of injuries requiring surgical intervention. In this respect, public health campaigns directed at parents, teachers, and children alike are crucial in reducing the severity of injuries. For children with penetrating or blunt injuries and manifestation of traumatic cataract, removal of the turbid lens can greatly improve visual acuity^8^. In addition, children with blunt injuries seemed to have better visual acuity than did those with penetrating injuries, both before and after cataract surgery.

To conclude, boys are more likely to suffer from traumatic cataract than are girls, with 2–8 years being the age group at highest risk of ocular injury, in the Chinese pediatric population. The major injury-causing object in all age groups was scissors, which should be placed out of reach of children, especially preschoolers. Cataract surgery can greatly improve the visual acuity of children with traumatic cataract. However, as all of the pediatric ocular injuries seen in this study could have been prevented, guardians or caretakers can better protect children from ocular injuries by becoming more aware of the risks associated with injury-causing objects.

## Methods

The Institutional Review Board of the Eye and Ear, Nose and Throat Hospital of Fudan University, Shanghai, China, approved this retrospective cross-sectional study affiliated with the Shanghai Pediatric Cataract Study (registered at www.clinicaltrials.gov, accession number NCT03063216). All procedures were conducted according to the tenets of the Declaration of Helsinki. Written informed consent forms were acquired from the guardians of the children for use of their medical data for research purposes.

### Review of Patient and Clinical Records

We conducted a retrospective chart review of all patients less than 18 years of age who underwent lens surgery for traumatic cataract at our hospital between March 2009 and March 2014. Those with complete medical history records were eligible for inclusion. Exclusion criteria were as follows: (1) a pre-existing ocular disease such as corneal opacity, congenital glaucoma, or optic atrophy and (2) a history of ocular surgery for oculopathies other than injury.

Personal information and medical data were documented and analyzed, including age, sex, laterality, mechanism of injury, causative agent, extent of the injury, interval between injury and first surgical intervention, and preoperative and postoperative visual acuity. The postoperative follow-up time was at least 1 month after the surgery. An international visual acuity measurement standard was used to examine the UCVA of children older than 3 years of age both before and after the cataract surgery, and the decimal notation of visual acuity was used for subsequent analyses. For children under 3 years old, visual acuity was assessed by examining their ability to follow a light stimulus. However, due to the nature of children, not all were able to cooperate in this examination. Only children with both preoperative and postoperative visual acuity data were selected for further analysis of visual acuity.

Preoperative examinations of traumatic cataract children included UCVA measurements (using an international visual acuity measurement standard), slit-lamp examination, axial length measurement in intraocular lens implantation cases, B-scan ultrasonography to examine posterior segment injuries, and X-ray examination to localize the intraocular foreign body in cases of open globe injury.

### Statistical analysis

Student’s t test was utilized to compare continuous variables and the chi-square test for categorical variables. Significance was set at P < 0.05. All statistical analyses were carried out using IBM^®^ SPSS^®^ statistics software, version 22 (IBM Corp., Armonk, NY, USA).
